# Periodontal Ligament, Cementum, and Alveolar Bone in the Oldest Herbivorous Tetrapods, and Their Evolutionary Significance

**DOI:** 10.1371/journal.pone.0074697

**Published:** 2013-09-04

**Authors:** Aaron R. H. LeBlanc, Robert R. Reisz

**Affiliations:** Department of Biology, University of Toronto Mississauga, Mississauga, Ontario, Canada; Team 'Evo-Devo of Vertebrate Dentition', France

## Abstract

Tooth implantation provides important phylogenetic and functional information about the dentitions of amniotes. Traditionally, only mammals and crocodilians have been considered truly thecodont, because their tooth roots are coated in layers of cementum for anchorage of the periodontal ligament, which is in turn attached to the bone lining the alveolus, the alveolar bone. The histological properties and developmental origins of these three periodontal tissues have been studied extensively in mammals and crocodilians, but the identities of the periodontal tissues in other amniotes remain poorly studied. Early work on dental histology of basal amniotes concluded that most possess a simplified tooth attachment in which the tooth root is ankylosed to a pedestal composed of “bone of attachment”, which is in turn fused to the jaw. More recent studies have concluded that stereotypically thecodont tissues are also present in non-mammalian, non-crocodilian amniotes, but these studies were limited to crown groups or secondarily aquatic reptiles. As the sister group to Amniota, and the first tetrapods to exhibit dental occlusion, diadectids are the ideal candidates for studies of dental evolution among terrestrial vertebrates because they can be used to test hypotheses of development and homology in deep time. Our study of Permo-Carboniferous diadectid tetrapod teeth and dental tissues reveal the presence of two types of cementum, periodontal ligament, and alveolar bone, and therefore the earliest record of true thecodonty in a tetrapod. These discoveries in a stem amniote allow us to hypothesize that the ability to produce the tissues that characterize thecodonty in mammals and crocodilians is very ancient and plesiomorphic for Amniota. Consequently, all other forms of tooth implantation in crown amniotes are derived arrangements of one or more of these periodontal tissues and not simply ankylosis of teeth to the jaw by plesiomorphically retaining “bone of attachment”, as previously suggested.

## Introduction

Tooth implantation is an important criterion for interpreting evolutionary events in major groups of tetrapods. The geometry of the attachment site of the tooth to the jaw has been used in phylogenetic reconstructions of lissamphibians [1], snakes and other squamates [2], [3] and mammals [4]. In general, three types of implantation are recognized: acrodonty (a tooth is attached to the apex of the jaw), pleurodonty (a tooth is attached to the lingual surface of the jaw), and thecodonty (a tooth is set into a deep socket in the jaw). Although these categories are convenient for partitioning tooth implantation into discrete types, or even character states, these three arrangements do not encompass the total diversity of ways in which teeth are implanted and attached to the jaws of tetrapods [4]. Some authors have proposed additional categories that take into account the geometry of implantation, as well as the nature of attachment of the tooth to the jaw in order to provide more specific classifications [4]. These classifications, however, have led to ambiguous and often conflicting interpretations of tooth implantation, particularly in extinct taxa [5], [6]. A more detailed and consistent definition of tooth implantation categories can be formulated at the histological level, which can be done for both extinct and extant groups [2], [7], [8].

**Figure 1:**
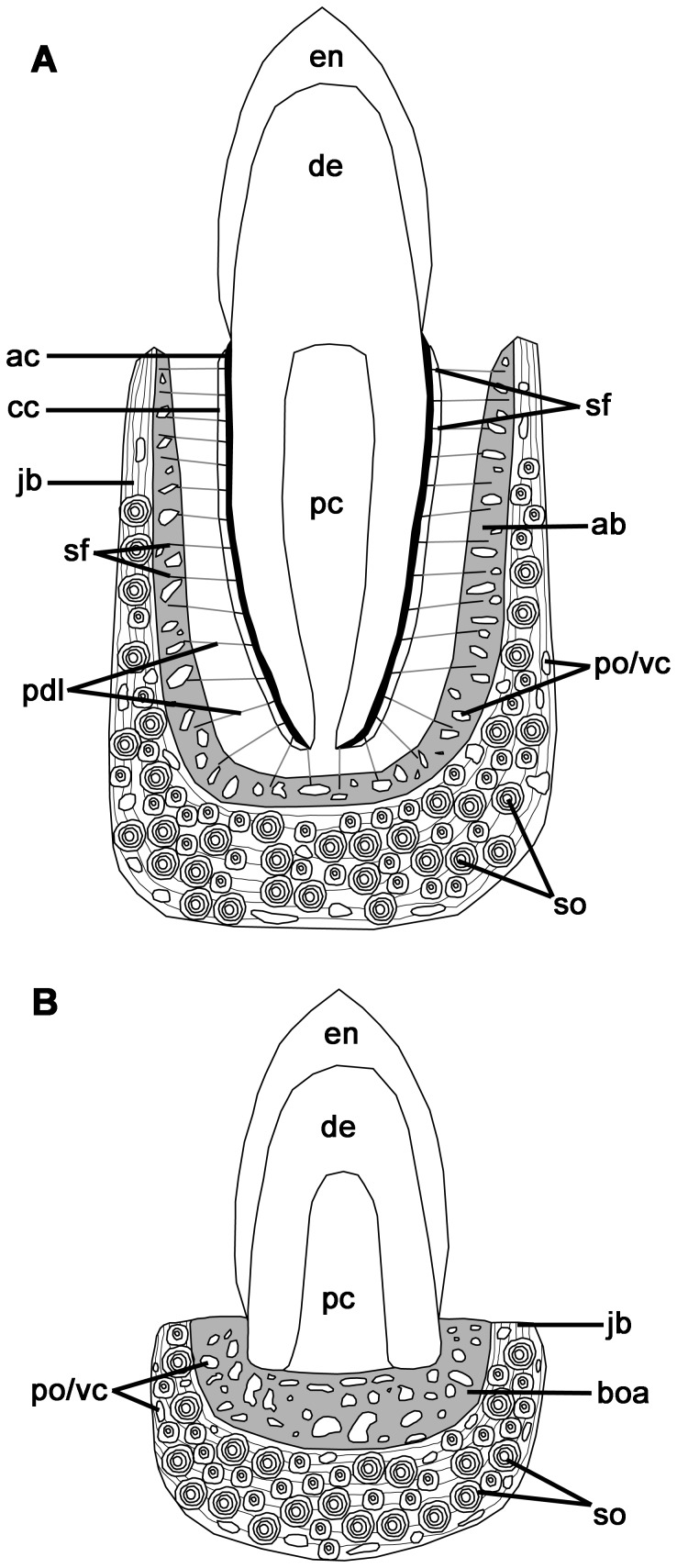
Comparisons of the tooth tissues in a generalized thecodont and subthecodont condition. A: thecodont tooth implantation in labiolingual section. B: subthecodont tooth implantation in labiolingual section. ab, alveolar bone; ac, acellular cementum; boa, bone of attachment; cc, cellular cementum; de, dentine; en, enamel; jb, jawbone; pc, pulp cavity; pdl, periodontal ligament; po, primary osteon; sf, Sharpey's fibers; so, secondary osteon; vc, vascular canal.

The histological properties of the tissues that attach a tooth to the jaw are well known for mammals and crocodilians, the two groups considered to exhibit true thecodonty [9]–[11]. As such, thecodonty is considered to be the most histologically complex, and hence the most derived form of implantation: the tooth root is coated in cementum, providing an attachment site for the periodontal ligament, which is in turn anchored to the alveolar bone that forms the tooth socket (Figure 1A). The cementum layers can be acellular or cellular and provide sites of attachment for the principal periodontal ligament fibers (Figure 1A). The periodontal ligament is mainly composed of an unmineralized network of collagen fibers, which serves multiple purposes, particularly in mammals: (1) it provides a flexible attachment of the tooth to the alveolar bone; (2) it facilitates post-eruptive tooth movement [12]; and (3) it serves as a sensory system to aid in proper positioning of the jaws during mastication [12]. The portion of the periodontal ligament that is embedded in the root cementum and in the alveolar bone is composed of Sharpey's fibers, which are completely or partially mineralized collagen fibers. In addition to possessing a ligamentous tooth attachment, mammals and crocodilians possess true tooth sockets, because the sockets are composed of alveolar bone. Alveolar bone is a vascularized bone tissue, with a matrix composed of dense Haversian bone (typical of mammals), or a loosely packed, woven-fiber matrix (more typical of crocodilians, but also found in mammals) [9]. Regardless of the matrix type, the alveolar bone forming the inner boundaries of the alveolus are lined with Sharpey's fibers, which mark the points of insertion for the fibers of the periodontal ligament (Figure 1A). Developmentally, all of these periodontal tissues are derived from the dental follicle, an aggregate of cells of ectomesenchymal origin that surrounds the developing tooth bud [13], [14].

By comparison, the teeth of non-mammalian and non-crocodilian tetrapods are usually ankylosed to the jaw [4], [7], [15]. The dentine of the tooth root is fused to a pedestal of bone that has been historically termed “bone of attachment”, which is in turn fused to the bone of the jaw (Figure 1B). Different proportions and arrangements of “bone of attachment” in relation to the tooth base and the jaw define the conventional categories of tooth implantation in non-mammalian and non-crocodilian taxa [16]. In general, “bone of attachment” is a term reserved for the vascularized bone tissue that surrounds the base of the tooth in non-mammalian and non-crocodilian tetrapods, and the developmental origins of this tissue are unclear.

**Figure 2:**
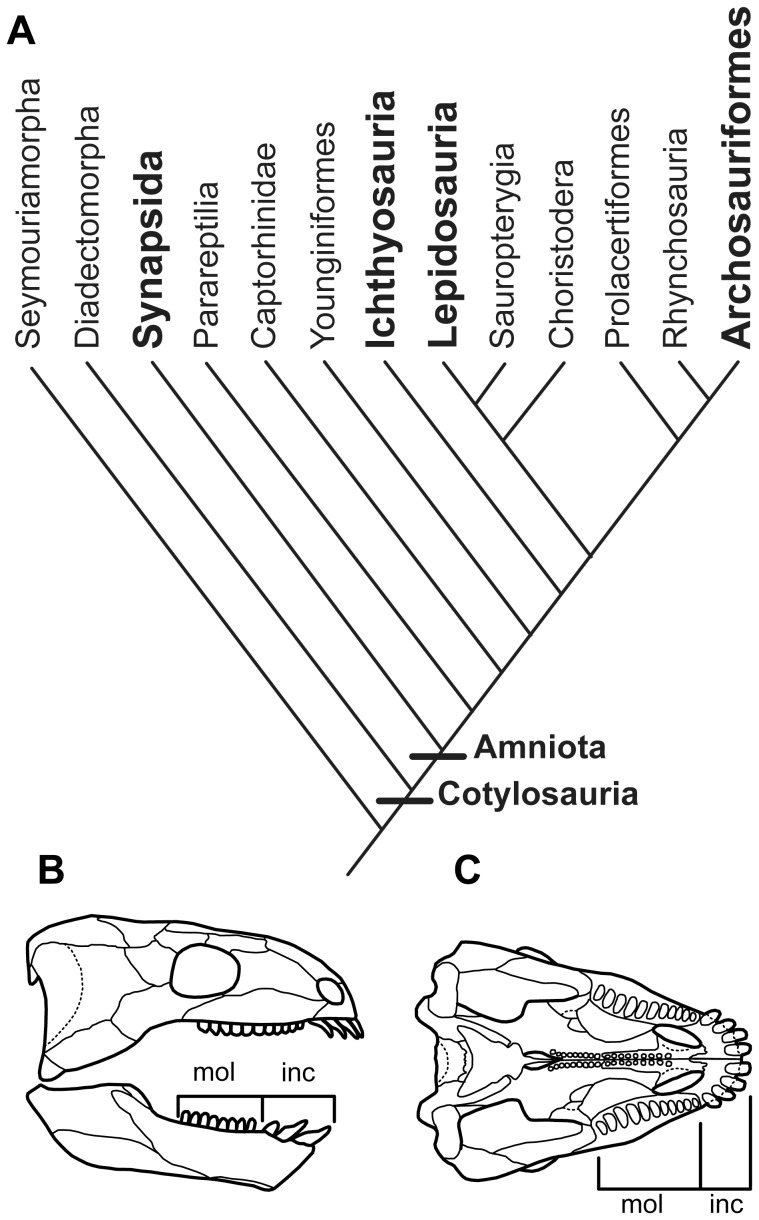
Phylogenetic position of Diadectomorpha and reconstruction of a diadectid skull. A: cladogram of stem and crown amniotes that are discussed. Modified from Maxwell, Caldwell, and Lamoureux [30]. Bolded terminal taxa are those that have representatives that possess alveolar bone and cementum. B: reconstruction of a diadectid skull in lateral view. Modified from Reisz [26]. Note the presence of anterior incisiform and posterior molariform teeth. C: reconstruction of a diadectid skull in ventral view. Modified from Reisz [26]. inc, incisiform teeth; mol, molariform teeth.

The difference in the modes of tooth attachment between mammals, crocodilians, and “lower” amniotes has been the subject of histological studies for decades [7], [15], [16]. More recent paleontological [2], [7], [8], [17]–[20] and developmental [16], [21]–[23] studies of amniotes have suggested that cementum, alveolar bone, and the periodontal ligament are present in some capacity even in non-mammalian and non-crocodilian amniotes (Figure 2A). Much of this recent work in dental histology, however, has been limited to studies of mammals, squamates [3], [7], extant crocodilians [11], [21], and ichthyosaurs [8], [24], and has not addressed the issue of homology of these tissues at the level of Amniota.

The present study expands the scope of dental tissue analyses to the stem amniotes, the diadectomorphs. Diadectomorpha occupy a uniquely important phylogenetic and paleoecological position in tetrapod evolution (Figure 2A), as the sister taxon to Amniota [25], [26]. Their fossil remains are known from Upper Pennsylvanian (approximately 305 million years ago) to Lower Permian (289 million years ago) localities in the United States and Europe [27]. Among diadectomorphs, the Diadectidae include the oldest known high fiber herbivores, the oldest known evidence of marked heterodonty (Figure 2B, 2C), and the oldest known evidence of dental occlusion and hence extensive oral processing in a tetrapod [26]. Diadectids are therefore ideal candidates for studies of tooth attachment at the beginning of amniote evolution, and for determining the primitive pattern for Amniota.

## Materials and Methods

Permission was obtained from all of the relevant institutions (Texas Memorial Museum in Austin, Texas; the Harvard University Museum of Comparative Zoology in Cambridge, Massachusetts; Royal Ontario Museum in Toronto, Canada) to access and work on the specimens that have been borrowed from their collections. All specimens were loaned to R. R. Reisz with permission for preparation and thin-sectioning.

Three specimens of the diadectid genus *Diadectes* from the Lower Permian of Texas accessioned at the Texas Memorial Museum in Austin, Texas (TMM 43628-3) and the Harvard University Museum of Comparative Zoology in Cambridge, Massachusetts MCZ 7871, 7874) were thin-sectioned. Additionally, an isolated incisiform tooth of a diadectid from the Lower Permian of Oklahoma, housed at the Royal Ontario Museum in Toronto, Canada (ROM 65911), and a single fragment of a left mandible of a horse (*Equus* sp., ROM 33036) from the Pleistocene of Florida were thin-sectioned for histological examination. Specimens were embedded in Castolite AP polyester resin, placed under vacuum and left to dry for 24 hours before being cut using a Buehler Isomet 1000 wafer blade low-speed saw. Specimens were mounted to glass slides using Scotch-Weld SF-100 cyanoacrylate or Hillquist epoxy resin. Specimens were ground down to approximately 180 µm thick using a Hillquist grinding cup, then ground by hand using progressively finer grits of silicon carbide powder. Some specimens were polished using one-micron grit aluminum oxide powder. Specimens were photographed using a Nikon DS-Fi1 camera mounted to a Nikon AZ-100 microscope fitted with crossed-polarizing and lambda filters, and an oblique illumination slider. A Meiji MT-9300 petrographic microscope fitted with crossed-polarizing and first-order red filters was also used for higher magnification images. Images were processed using Nikon NIS-Elements (Basic Research) imaging software registered to D. C. Evans of the Royal Ontario Museum and NIS-Elements (Basic Research) registered to R. R. Reisz of the University of Toronto Mississauga.

## Results

**Figure 3:**
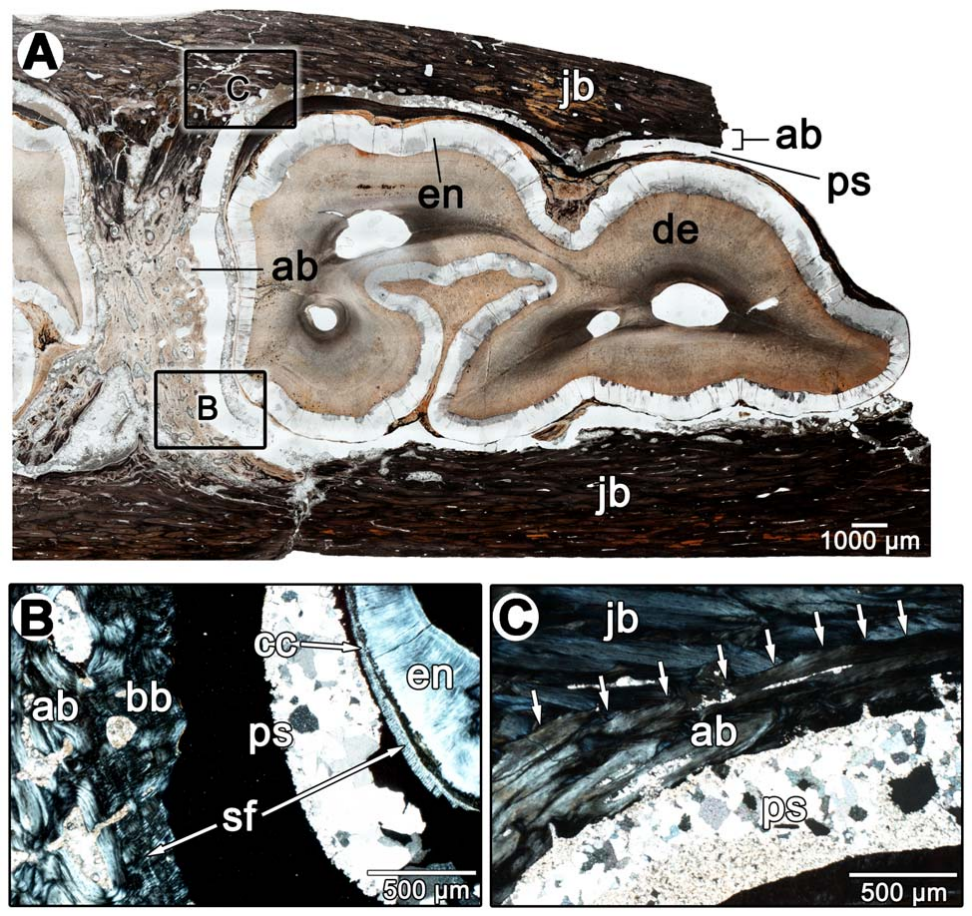
Dental histology of *Equus* sp. (ROM 33036). A: overview image of a cross-section through the partial left mandible of ROM 33036, taken parallel to the tooth row and bisecting a molariform tooth. B: closeup of the periodontal region in A under cross-polarized light. Note the presence of Sharpey's fibers on either side of the periodontal space, indicating the presence of a periodontal ligament that has since disintegrated. C: closeup of the alveolar bone in A under cross-polarized light. Arrows highlight the position of a reversal line between the alveolar bone and the jawbone. ab, alveolar bone; bb, bundle bone layer within the alveolar bone; cc, cellular cementum; de, dentine; en, enamel; jb, jawbone; ps, periodontal space; sf, Sharpey's fibers.

Taphonomic and diagenetic processes can alter tissues of animals. We therefore propose to use a fossil horse specimen (*Equus* sp., ROM 33036) from the Pleistocene of Florida, USA for comparisons with the tissues observed in diadectids in order to ensure consistent definitions of the individual tooth and periodontal tissues across fossil taxa, using the mammalian condition as a reference. This approach allows us to understand how the mammalian condition for tooth implantation and attachment is modified after fossilization, and therefore reconstruct the tissues that originally existed in the diadectid jaw, before fossilization. All periodontal tissues that are present in extant horses are readily identifiable in cross-sections of ROM 33036, including the mineralized portion of the periodontal ligament (Figure 3). As hypsodont (high-crowned) ungulates, horses possess highly infolded and extensive layers of dentine, enamel, and cementum that form significant portions of the tooth [28]. The enamel, typically associated with the portion of the crown that is above the jawbone and gum line in brachydont (short-crowned) mammals, is found in cross-sections that are taken from deep within the jaw in *Equus* sp. (Figure 3A, 3B). Layers of cellular cementum coat the periphery of the tooth and are external to the enamel. Normally this tissue is situated external to the dentine of the tooth root in brachydont mammals [29]. Under cross-polarized light, the cellular cementum contains numerous Sharpey's fibers, which extend radially around the tooth (Figure 3B). These Sharpey's fibers mark the insertion points for the periodontal ligament into the cellular cementum. The unmineralized portion of the periodontal ligament would have occupied the area external to the cellular cementum. In its place is a void in fossilized specimens, which is partially or completely infilled with diagenetic minerals in different parts of the alveoli in the *Equus* sp. material (Fig. 3A, 3B). Alveolar bone forms the inner walls of the tooth socket (Figure 3B, 3C). This tissue is separated from the bone of the jaw by a reversal line in some areas, which indicates the farthest extent to which bone of the jaw had been resorbed to accommodate the alveolar bone (Figure 3C). Alveolar bone in *Equus* sp. is highly remodeled and highly vascularized. Secondary osteons are prevalent throughout the alveolar bone, but are particularly abundant in more external layers (those closest to the bone of the jaw). Primary bone tissues are confined to the regions that are closest to the periodontal space. These internal layers are composed of lamellar bone with abundant Sharpey's fibers (Figure 3B). This tissue, termed bundle bone, provides anchorage for the periodontal ligament into the walls of the alveolus [29].

**Figure 4:**
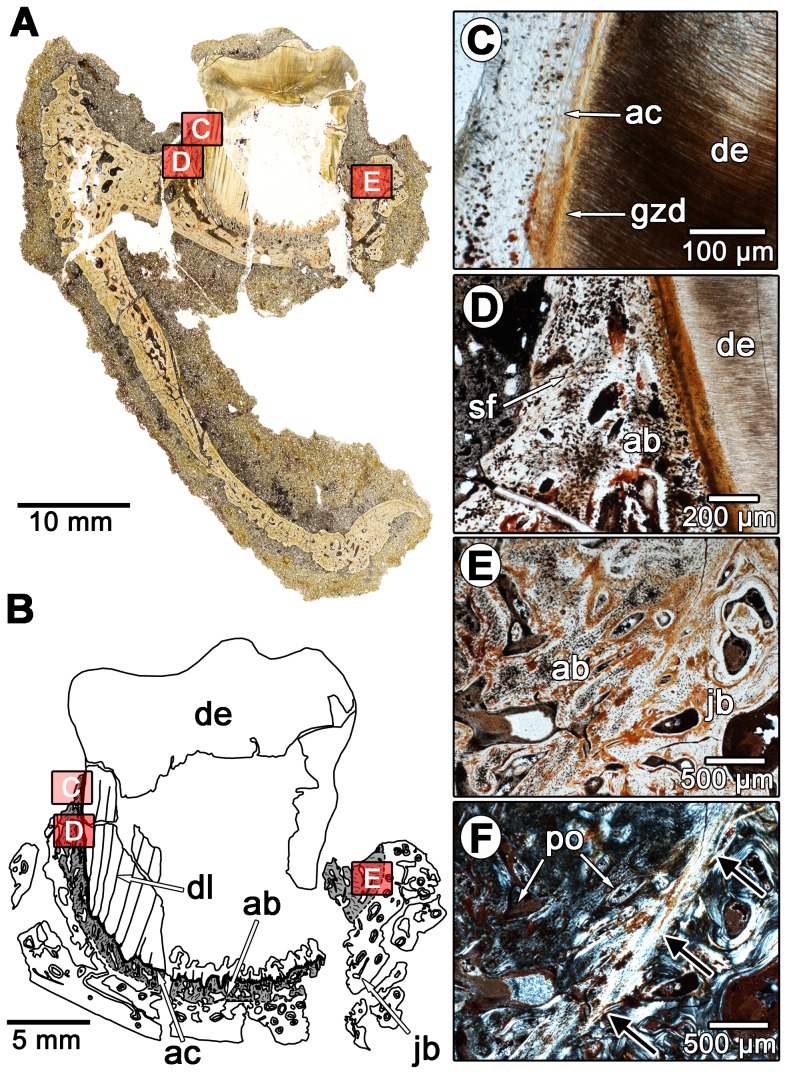
Dental histology of a molariform tooth in *Diadectes* sp. (MCZ 7874) from the Permian of Texas. A: overview image of a labiolingual section of a molariform tooth from an isolated dentary. The red boxes correspond to the positions of images C–F. B: interpretation of the dental tissues in A. The alveolar bone is shaded grey. The red boxes correspond to the positions of images C–F. C: closeup of the tooth root from the labiolingual section in A. D: closeup of the tooth root and adjacent alveolar bone from the labiolingual section in A. Note the absence of a periodontal space between the alveolar bone and the tooth root. E: closeup of the alveolar bone and the adjacent jawbone from the labiolingual section in A. Note the darker color of the alveolar bone, which is a result of a high density of Sharpey's fibers. F: same image as in E, but under cross-polarized light. Note the reversal line highlighted by the black arrows, which separates the woven bone of the alveolus from the Haversian bone of the jaw. ab, alveolar bone; ac, acellular cementum; de, dentine; dl, dentine lamellae; gzd, globular zone of dentine; jb, jawbone; po, primary osteon; sf, Sharpey's fibers.

**Figure 5:**
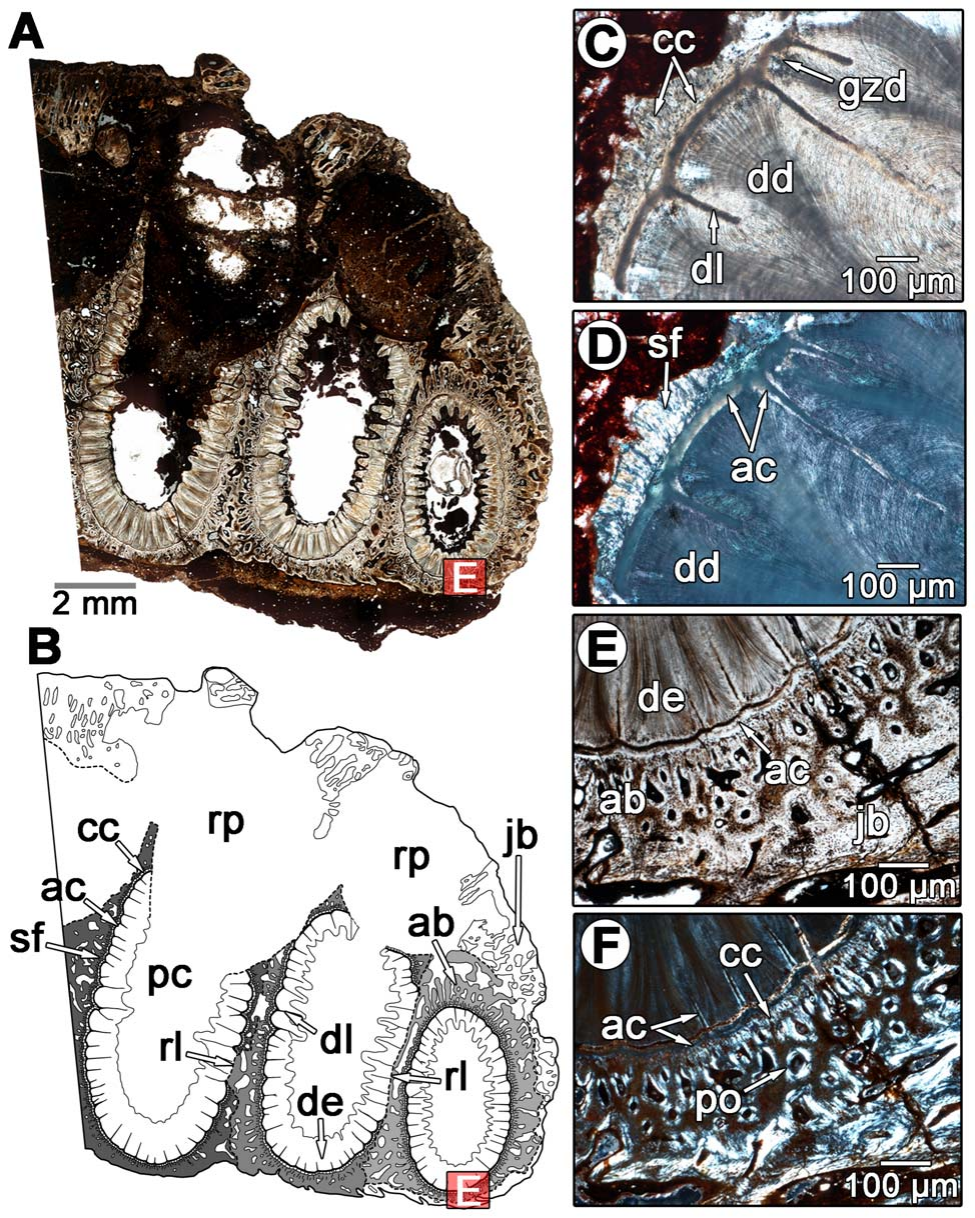
Cross-sectional views of the molariform teeth of *Diadectes* sp. from the Permian of Texas. A: overview image of a cross-section through the roots of three molariform teeth of *Diadectes* sp. (MCZ 7871). Note the presence of large resorption pits lingual to the tooth roots, which have invaded the pulp cavities of two of the teeth. Red box indicates position of image E. B: Interpretation of the dental tissues in A. Grey areas indicate alveolar bone of each associated root, with darker shades of grey indicating older generations of alveolar bone. Dashed lines indicate reversal lines. C: cross-section of a large diadectid molariform tooth root (TMM 43628-3), possessing thick layers of cementum and clear dentine infoldings. D: same image as in C, but under cross-polarized light. Note the distinct boundary between the acellular cementum and the dentine, and the extension of the acellular cementum into the cores of some of the dentine infoldings. Also note the presence of Sharpey's fibers in the cellular cementum. E: closeup of a *Diadectes* tooth root and alveolar bone from image A. F: same image as E, but under cross-polarized light. Note the presence of acellular cementum within the cores of some of the dentine infoldings and the birefringence of the cellular cementum layer. ab, alveolar bone; ac, acellular cementum; cc, cellular cementum; dd, dark dentine; de, dentine; dl, dentine lamella; gzd, globular zone of dentine; jb, jawbone; pc, pulp cavity; po, primary osteon; rl, reversal line; rp, resorption pit; sf, Sharpey's fibers.

The thin sections of diadectid teeth reveal a similarly complex organization of dental and periodontal tissues. The molariform and incisiform teeth of diadectids are housed in deep sockets that are lined with a highly vascularized bone tissue (Figure 4A, 4B). The dentine portion of the tooth is composed of typical orthodentine; long tubules that would have housed the odontoblast processes and nerve filaments extend radially from the pulp cavity towards the external surface of the tooth (Figure 4C, 4D). At the outer edge of the orthodentine layer, the tubules terminate within a zone of poorly mineralized, irregular dentine known as the globular zone, similar to other tetrapods [8]. The outer walls of the tooth roots possess longitudinal striations (Figure 4B). Cross-sectional views of the tooth roots show that these striations form plicidentine (Figure 5). In general, plicidentine is defined as infoldings of the dentine portion of the root towards the pulp cavity [30]. The presence of plicidentine in diadectomorphs was first reported for the basal diadectomorph *Limnoscelis* [31], but is described histologically in diadectomorphs for the first time here. Plicidentine in diadectids consists of straight, radial infoldings that occasionally branch as they extend towards the pulp cavity in cross-section (Figure 5A, 5B). The infoldings consist of orthodentine lamellae [30]: tight infoldings of the orthodentine that are not invaded by the external layer of vascularized bony tissue (Figure 5C, 5D). The orthodentine layers between the lamellae form “dark dentine”, dense zones of dentine tubules that are the result of the clustering of odontoblasts in these regions during the deposition of the dentine matrix [30], [32]. The infoldings are relatively numerous, with a single tooth in one of the larger specimens (TMM 43628-3) possessing at least 45 dentine lamellae in cross-section.

External to the globular zone of dentine is a 50 µm thick band of tissue in the root portion of the tooth (Figure 4C). This tissue lacks cell spaces and produces a distinct extinction pattern under crossed-polarized light, and in strong contrast to the adjacent globular zone and more external layers (Figure 5D–5F). Topologically and histologically, this tissue is identifiable as acellular cementum, as in mammals, crocodilians, and marine reptiles [2], [3], [8], [11], [17]. In diadectids the acellular cementum coats the root orthodentine and even extends into the cores of some of the larger dentine lamellae (Figure 5C, 5D).

**Figure 6:**
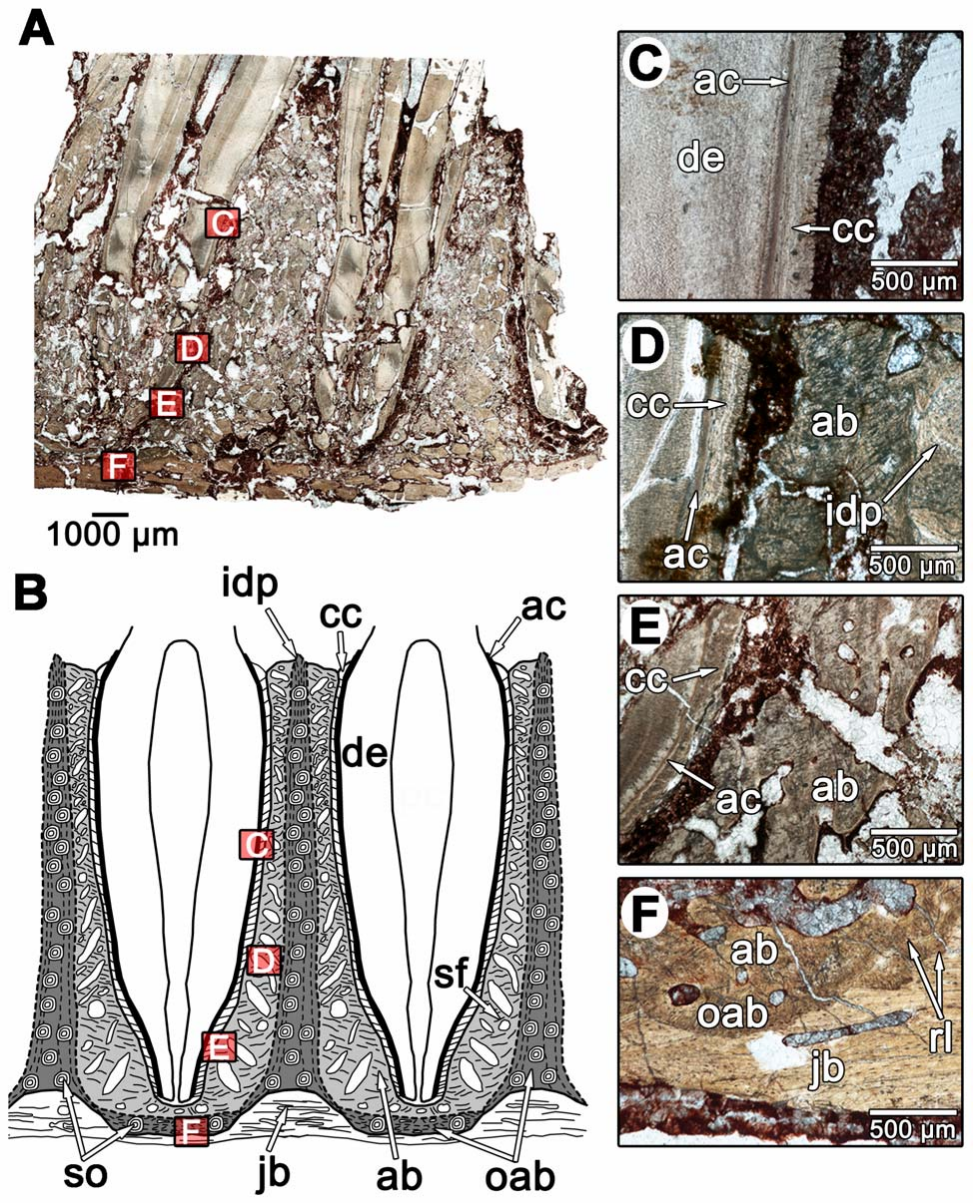
Longitudinal section of the incisiform teeth of a diadectid (TMM 43628-3) from the Lower Permian of Texas. A: Overview image of a longitudinal section through two complete tooth roots. Red boxes correspond to images C–F. B: interpretation of the arrangements of the periodontal tissues of diadectids in longitudinal section. Grey areas indicate alveolar bone and darker areas indicate the presence of older generations of alveolar bone from previous teeth. Red boxes correspond to the positions from which images C–F were taken. C: closeup of the root of one of the teeth from image A. Note the presence of acellular and cellular cementum, as well as an apparent periodontal space. D: closeup of of the tooth root and socket of one of the teeth from image A. The darker coloration of the alveolar bone is due to the presence of Sharpey's fibers. Note the presence of another bone tissue forming the wall of the alveolus, here referred to as the interdental plate. E: closeup of the base of a tooth root and alveolar bone from image A. F: closeup of the base of the alveolus of one of the teeth from image A. Note the presence of multiple generations of alveolar bone forming the floor of the alveolus. ab, alveolar bone; ac, acellular cementum; cc, cellular cementum; de, dentine; idp, interdental plate; jb, jawbone; oab, old generation of alveolar bone; pc, pulp cavity; rl, reversal line; sf, Sharpey's fibers; so, secondary osteons.

**Figure 7:**
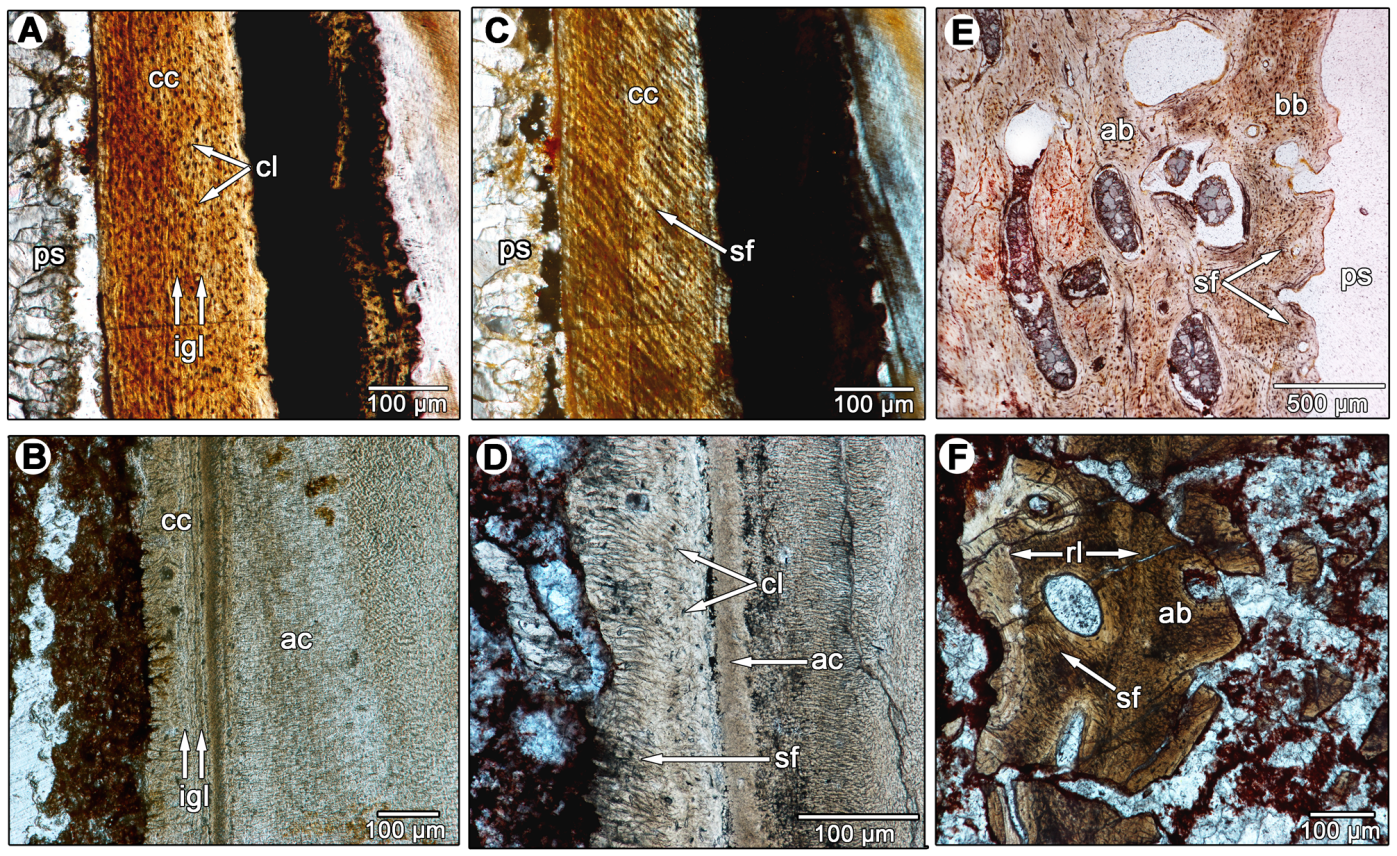
Comparisons of the periodontal tissues between diadectids and a fossil horse. A: longitudinal section of cellular cementum in a molariform tooth of *Equus* sp. (ROM 33036) under normal light. B: longitudinal section of the acellular and cellular cementum of a diadectid molariform tooth (TMM 43628-3). Image was taken using an oblique illumination slider to highlight the incremental growth lines in the cellular cementum. C: longitudinal section of cellular cementum in *Equus* sp. (ROM 33036) under cross-polarized light. Note the extensive network of parallel Sharpey's fibers that mark the insertions of the periodontal ligament. D: closeup of the acellular and cellular cementum of a diadectid tooth (TMM 43628-3). Note the presence of a network of parallel Sharpey's fibers that mark the insertions of the periodontal ligament. E: closeup of the alveolar bone of *Equus* sp. (ROM 33036) in cross-section. Note the presence of Sharpey's fibers in the alveolar bone layers that border the periodontal space. F: closeup of the alveolar bone of a diadectid (TMM 43628-3) in cross-section. Note the presence of dense networks of Sharpey's fibers in successive layers of alveolar bone. A reversal line separates each layer of alveolar bone. ab, alveolar bone; ac, acellular cementum; bb, bundle bone layer of the alveolar bone; cc, cellular cementum; cl, cementocyte lacunae; igl, incremental growth lines in the cementum; ps, periodontal space; rl, reversal line; sf, Sharpey's fibers.

Lining the acellular cementum layer is a wavy band (approximately 50–100 µm thick) of cellular bone-like tissue that possesses abundant incremental lines and lacks vascular spaces (Figure 6A–6E). In longitudinal section, the wavy incremental lines extend parallel to the apical-occlusal axis of the tooth root, similar to the incremental growth lines in the cellular cementum of mammals [33], including the fossil specimen of *Equus* sp. (Figure 7A, 7B). This striking similarity between the cellular cementum of mammals and non-mammalian amniotes [8], [11], [24] and the incrementally banded layer of diadectids allow us to identify the latter as cellular cementum. The diadectid cellular cementum has a second layer external to the incremental bands that does not possess any clear incremental banding, and is characterized by high densities of Sharpey's fibers that are oriented nearly perpendicular to the apical-occlusal axis of the tooth root in longitudinal section (Figure 7C). We interpret this external layer as the point of attachment of the periodontal ligament to the cellular cementum, which is similar to the condition in *Equus* sp. (Figure 7D). This external layer of cellular cementum in diadectids is as thick as, or thicker than the internal, incrementally banded cellular cementum layer.

The major component of the tooth socket in diadectids is a vascularized bony tissue that we identify here as alveolar bone, situated between the cementum coating the tooth root and the bone of the jaw (Figures 4–6). This tissue is clearly distinguished from the remodeled bone of the jaw by a reversal line (Figures 4F, 6F), and primary osteons and simple vascular canals are present throughout in all of the diadectid specimens that we examined. The matrix consists of woven-fiber bone, a rapidly deposited bone matrix that is characterized by random orientations of the collagen fiber matrix [3]. In longitudinal section, the bases and walls of some of the alveoli are formed by previous generations of this woven-fiber bone, and are separated from more recent generations by reversal lines (Figure 6D, 6F). This would suggest that most of this tissue was shed along with the tooth during each tooth replacement event, unlike the condition in crocodilians, but similar to mammals [11], a pattern that is consistent with what other authors have reported in the alveolar bone of ichthyosaurs, mosasaurs, and snakes [3], [8], [34]. This alveolar bone (Figures 4B, 5B, 6B) shows evidence of repeated resorption and re-deposition, indicating that its formation is closely linked with the development of each new tooth, a well known developmental characteristic of alveolar bone [10], [35].

A periodontal space is present in some of the diadectid specimens, but absent in most (Figures 4–6). The diadectid specimen from the Mud Hill locality of Texas (TMM 43628-3) appears to have periodontal spaces, as revealed in thin section, but this feature may be diagenetic (Figure 6C–6E). However, the alveolar bone and cementum of all of the specimens possess abundant Sharpey's fibers, similar in orientation and density to those in the alveolar bone and cementum of mammals (Figures 4D, 4E, 5, 6, 7F). Dense networks of Sharpey's fibers extend towards the tooth root in all views. In *Equus* sp. (ROM 33036), Sharpey's fibers provide traces of the former position of the periodontal ligament between the cementum of the tooth and the alveolar bone (Figures 3B, 7C, 7E). Sharpey's fibers are thus important indicators of the presence of a periodontal ligament in mammalian and non-mammalian taxa [2], [3], [7], [8]. Sharpey's fibers are only known to exist at syndesmoses, sites of soft tissue attachment to bone, and in the portion of the periodontal ligament that contacts the alveolar bone and root cementum [36]. The presence of Sharpey's fibers throughout the alveolar bone and cellular cementum of diadectids and a lack of a periodontal space would suggest that even in those specimens that show an apparent ankylosis of the tooth to the socket, the ligament was present in some capacity. This condition is very similar to that seen in mosasauroid squamates, where a portion of the spongy bone-like tissue that connects the cementum of the roots of the teeth to the alveolar bone has been interpreted as a calcified periodontal ligament [7]. A similar interpretation is applicable to diadectids, but the boundary between alveolar bone and the mineralized periodontal ligament is difficult to define. Previous authors have had similar difficulty differentiating the two tissues because both can possess abundant Sharpey's fibers and high concentrations of vascular spaces [7].

**Figure 8:**
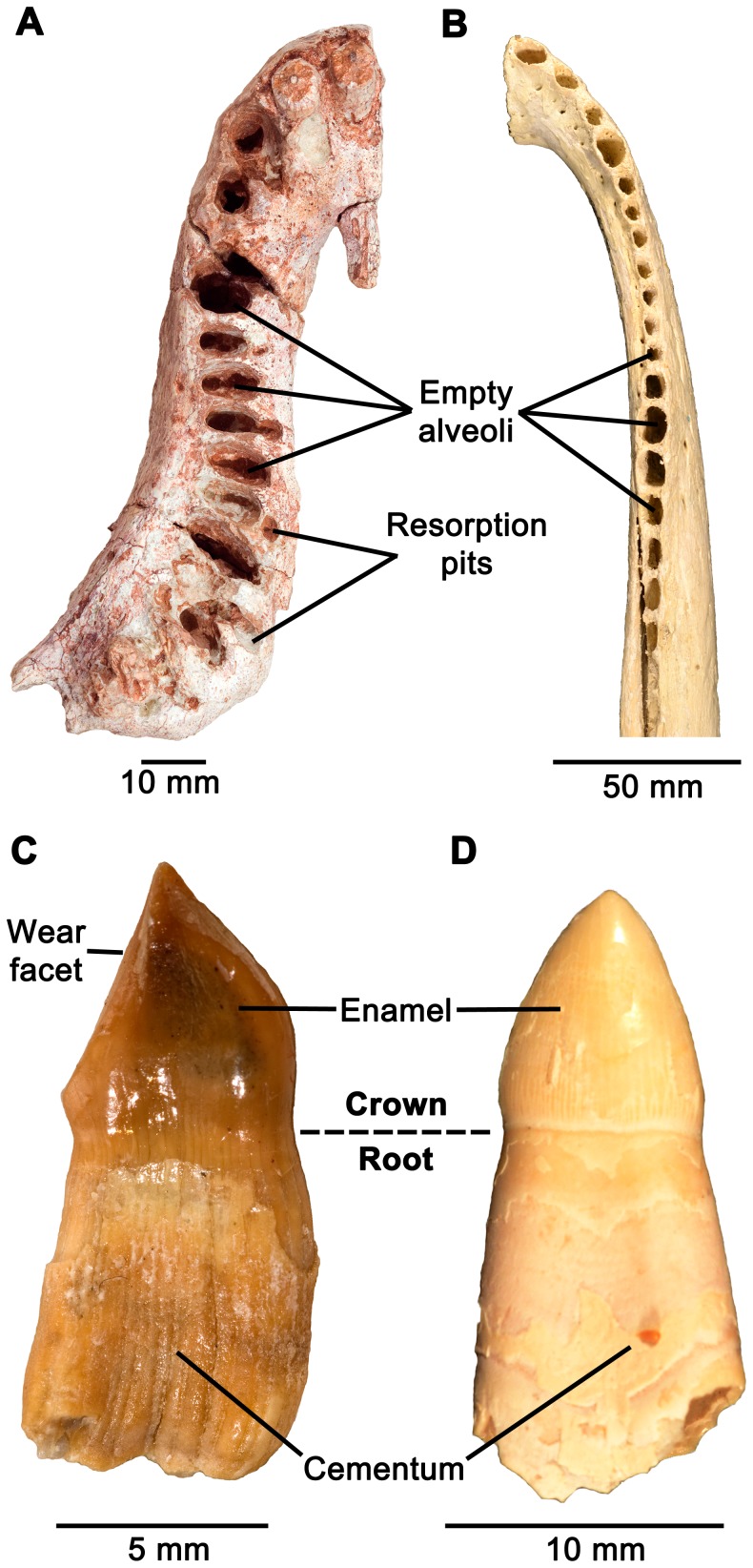
Taphonomic evidence for the presence of periodontal ligaments in diadectids and crocodilians. A: ventral view of a complete right upper jaw (premaxilla and maxilla) of a diadectid (TMM 43628-2) that exhibits post-mortem tooth loss. Nearly all of the teeth are interpreted as having been lost after the periodontal ligament had decomposed. B: dorsal view of a dentary of a modern *Alligator mississippiensis* (ROM 690) that exhibits post-mortem tooth loss. All of the teeth have fallen out as a result of a loss of the periodontal ligament after death. C: an isolated diadectid tooth with a complete root from the Lower Permian Dolese Brothers Quarry near Richards Spur, Oklahoma, part of a collection of isolated diadectid teeth from the Sam Noble Oklahoma Museum of Natural History in Normal, Oklahoma (OMNH 56872). The presence of a complete root and a worn crown suggest that this tooth was functional and was not shed from the jaw, but was lost post-mortem. Note the presence of a thick layer of cementum coating the root. D: an isolated tooth of *Alligator mississippiensis* (ROM 690) that has fallen out of the dentary due to the loss of the periodontal ligament.

**Figure 9:**
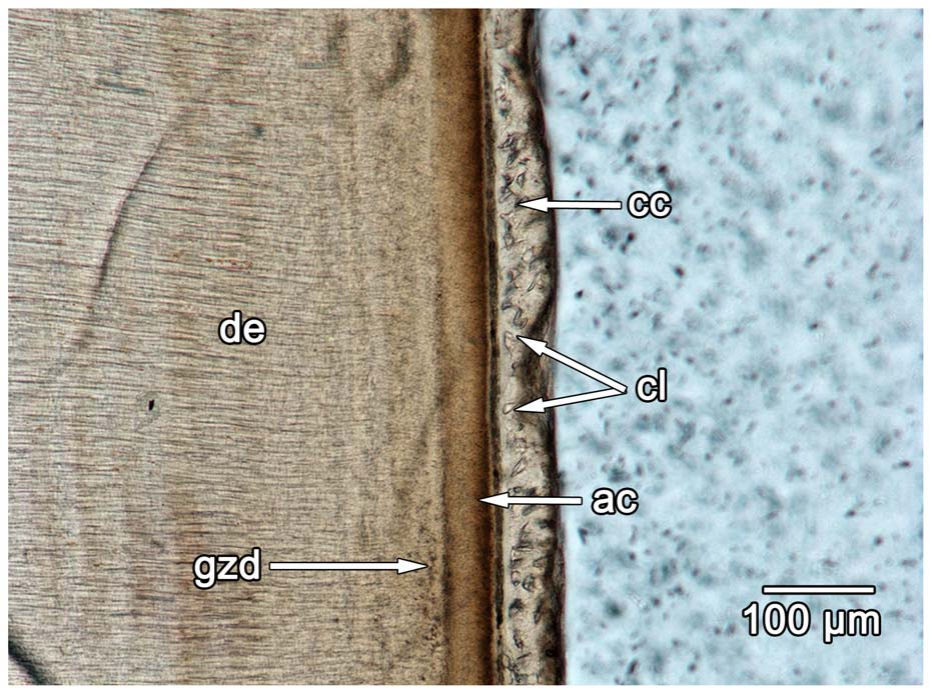
Longitudinal section of an isolated diadectid incisiform tooth (ROM 65911). ac, acellular cementum; cc, cellular cementum; cl, cementocyte lacunae; de, dentine; gzd, globular zone of dentine.

Examination of more complete jaws of diadectids provides additional taphonomic evidence that they possessed periodontal ligaments. A left dentary, and a right maxilla and premaxilla attributable to *Diadectes* (TMM 43628-2) from the Lower Permian Mud Hill locality of Texas exhibit an unusual taphonomic feature of the dentition. In both elements, nearly all of the teeth have been lost post-mortem (Figure 8A), and the empty alveoli do not contain any visible remnants of the tooth roots, indicating that the teeth were lost and not broken at their bases. This phenomenon is certainly not related to typical tooth replacement, because diadectids are known to have replaced their teeth in alternating waves, similar to iguanian lizards [37]. This replacement pattern would never result in such large gaps in the dentition, particularly in an herbivorous tetrapod that possessed a row of occluding molariform teeth for oral processing [26]. This loss is similar to the commonly seen phenomenon of post-mortem tooth loss in mammals and crocodilians, which is rare in lizards and other tetrapods with ankylosed teeth [38]. The periodontal ligament decays after death in mammals and crocodilians, leaving no soft tissue connection between the tooth root and the alveolus (Figure 8B), and teeth often fall out of the alveoli once the ligamentous attachment has decayed (Figure 8D). Similarly, diadectids that have lost significant numbers of teeth must have possessed a soft tissue attachment between the cementum coating the tooth root and the alveolar bone that decayed after the death of the individual. Finally, isolated diadectid teeth from the Lower Permian Richards Spur locality of Oklahoma show no evidence of being shed as a result of tooth replacement [39]. These isolated teeth possess complete, unresorbed roots, as well as wear facets, indicating that they were functional prior to falling out of the jaw (Figure 8C). As in extant mammals and crocodilians, the roots of these isolated diadectid teeth are coated in cementum, which would have provided anchorage for the periodontal ligament (Figure 8C, 8D). Interestingly, thin sections of an isolated incisiform tooth of a diadectid from Richards Spur (ROM 65911) show nearly identical arrangements of acellular and cellular cementum to those of crocodilian and mammalian teeth [11], [29], suggesting very similar modes of attachment of these teeth (Figure 9). Thus, both histological and taphonomic evidence indicate that diadectids must have possessed a ligamentous attachment at some point in the ontogeny of individual teeth, but may have become calcified at later stages.

## Discussion

### Tooth Attachment and Implantation in Diadectidae

Our histological analysis of the diadectid periodontium demonstrates that there is considerable complexity in the mode of tooth implantation and attachment, comparable to thecodonty in crocodilians and mammals. Numerous lines of evidence support our interpretation that tooth attachment was accomplished by a union of cementum to alveolar bone, via a periodontal ligament. Interestingly, diadectid teeth possess abundant infoldings of the dentine portions of the tooth roots (plicidentine) (Figure 5); however, these infoldings did not increase the surface area of attachment of the teeth to the jaw as they do in other tetrapods [30]. The infoldings of the dentine are expressed as thin longitudinal striations along the outer surfaces of the tooth roots (Figures 4B, 8C) that could not have appreciably increased the external surface area of the tooth root. This would suggest that plicidentine in diadectids may have served a purpose that is entirely different from that of labyrinthodont amphibians or modern varanid lizards [30].

Tooth implantation in diadectids has traditionally been referred to as a thecodont ankylosis [15], [37]. By virtue of the current classification of vertebrate tooth attachment [4], [7], this would imply that the teeth were deeply implanted and fused to the jaw through an intermediary layer of “bone of attachment” (Figure 1B). However, the distinction between the alveolar bone of mammals and crocodilians, and the “bone of attachment” of “lower” amniotes appears to be made on the basis of taxonomy rather than histology or developmental biology. Many authors have noted that there are very few histological, and no developmental characteristics of “bone of attachment” that distinguish this tissue from the periodontal tissues of mammals and crocodilians [2], [3], [34]. Furthermore, the term “bone of attachment” is misleading in this context, because it is unknown whether “bone of attachment” is homologous with cementum, alveolar bone, or both [3], [15], [40]. The vascularized bone that forms in the alveolus in diadectids, mammals, and crocodilians is a periodontal tissue distinct from cementum and the periodontal ligament. The developmental origins of this bone in mammals and crocodilians are clear, however: it is a product of the dental follicle, along with cementum and the periodontal ligament [11], [35]. As such, we refer to the vascularized bone tissue that lines the walls of the alveolus in diadectids and all amniotes as alveolar bone. In this way, we explicitly acknowledge its distinctiveness from cementum and the periodontal ligament, and its similarities to the mammalian and crocodilian socket bone.

**Figure 10:**
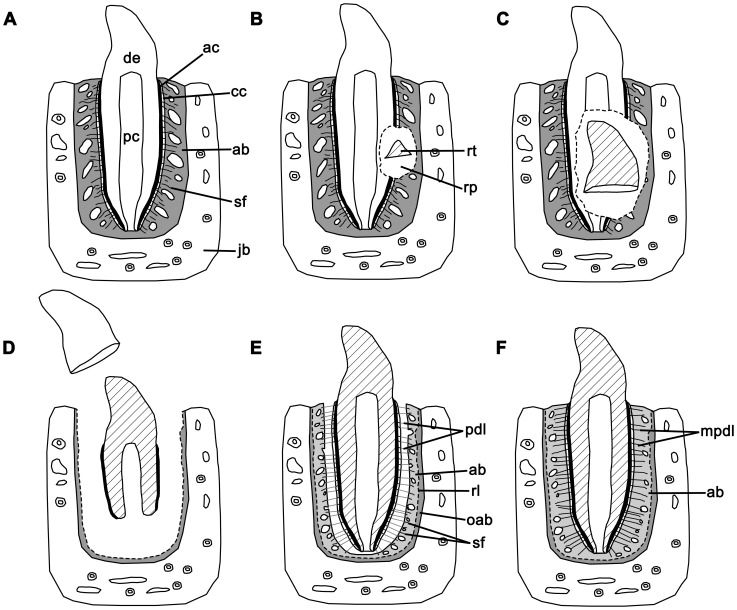
Interpretation of the development of the periodontal tissues in diadectids in a full tooth replacement cycle. A: the periodontal tissues of an incisiform tooth are fully developed; the tooth is bounded to the alveolus by a completely mineralized periodontal ligament and alveolar bone. B: a replacement tooth begins to form within a resorption pit lingual to the functional tooth, causing resorption of the surrounding dentine and periodontal tissues. C: the replacement tooth and resorption pit enlarge, invading the pulp cavity of the functional tooth. D: the functional tooth is shed and the replacement tooth begins to migrate into the oral cavity. Not all of the alveolar bone from the previous generation is resorbed. Root dentine and acellular cementum of the replacement tooth begin to form. E: The new tooth becomes functional and is suspended by an unmineralized periodontal ligament. The tooth root is coated in both acellular and cellular cementum. A new generation of alveolar bone overlies the previous layer. F: the fully mature tooth is firmly attached to the alveolus by a mineralized periodontal ligament. ab, alveolar bone; ac, acellular cementum; cc, cellular cementum; de, dentine; jb, jawbone; mpdl; mineralized periodontal ligament; oab, older generation of alveolar bone; pc, pulp cavity; pdl, periodontal ligament; rl, reversal line; rp, resorption pit; rt, replacement tooth; sf, Sharpey's fibers.

The thin sections taken at different points in the replacement cycle permit reconstruction of the development of the diadectid periodontium. The formation and development of the periodontium in diadectids were very similar to those of mammals and crocodilians. At the point in which all of the periodontal tissues were fully developed, the tooth was functional, deeply implanted into the alveolus, and firmly attached to the alveolar walls (Figure 10A). There was no periodontal space at this point. The tissue that occupied the area between the alveolar bone and the cellular cementum coating the tooth root was a vascularized, mineralized tissue that possessed abundant Sharpey's fibers. We have interpreted this tissue as a mineralized periodontal ligament. It is difficult to define the boundary between what we interpret as the mineralized periodontal ligament and the alveolar bone, because both of these tissues possess very high concentrations of Sharpey's fibers that obscure any tissue boundaries (Figure 6).

During the course of development of a new tooth, a replacement pit would form along the lingual side of the functional tooth root, causing resorption of root dentine, cementum, mineralized periodontal ligament, and alveolar bone (Figures 5A, 5F, 10B, 10C). Soon after the replacement pit had become large enough to invade the pulp cavity of the functional tooth, the tooth was shed (Figure 10D). At this stage, the replacement tooth was not yet functional and the alveolus was lined with remnants of the previous generation of alveolar bone. In thin section, reversal lines between each generation of alveolar bone mark the furthest extent to which bone resorption took place within the alveolus, prior to the formation of a new layer of alveolar bone (Figures 6B, 6D, 6F; 7F). At this stage, the replacement tooth possessed only a partially developed dentine root that was lined with acellular cementum (Figure 10D). This is inferred based on the development of replacement teeth in mammals and crocodilians [21], [29]. Pre-functional replacement teeth in mammals and crocodilians are coated in acellular cementum, whereas the cellular cementum is only formed once the tooth is functional [21], [29]. At the next stage, the replacement tooth reached its functional position, possessed an outer layer of cellular cementum, and was attached to a new layer of alveolar bone via a periodontal ligament (Figures 8C, 9, 10E). The arrangements of the acellular and cellular cementum in teeth from this stage are nearly identical to those of functional teeth in mammals and crocodilians (Figure 9). At this stage, tooth implantation was a thecodont gomphosis, because the tooth was ligamentously attached to alveolar bone. Finally, at the last stage, the periodontal ligament became mineralized, closing the periodontal space (Figures 6, 10F). Over multiple generations of alveolar bone resorption and re-deposition, the floors of the alveolus would consist of the remnants of multiple generations of alveolar bone. Similarly, the remains of successive generations of alveolar bone along the walls of the alveolus would produce the interdental plates that separate adjacent teeth [3], [34].

### Comments on the Evolution of Amniote Tooth Implantation and the Homologies of the Periodontal Tissues

Early comparative studies of amniote tooth implantation concluded that mammals and crocodilians are the only modern taxa to exhibit true thecodonty [15]. Histological and developmental properties of the periodontal tissues are nearly identical in the two distantly related groups [11]. Several authors have discovered more recently the presence of a tripartite periodontium, consisting of cementum, periodontal ligament and alveolar bone in fossil squamates and ichthyosaurs [2], [3], [8], [17]. Interestingly, recent studies of extant squamates have also shown that the stereotypically pleurodont mode of tooth implantations in *Varanus* and *Iguana* also consist of cementum and alveolar bone, albeit in different arrangements from those associated with a thecodont tooth implantation [7], [41]. These findings raise the possibility that the ability to produce the three periodontal tissues may be a synapomorphy of Amniota, or at least is a plesiomorphy for all amniote taxa that have been examined thus far. Our examination of the diadectid condition supports this hypothesis, but also adds additional information on the distribution of this feature within a more inclusive clade of tetrapods. The presence of acellular and cellular cementum, a periodontal ligament, and alveolar bone in Diadectidae suggests that the ability to produce a tripartite periodontium is not a synapomorphy of Amniota, but can now be extended to all Cotylosauria (Diadectomorpha + Amniota). This has important implications for how the evolution of tooth implantation is interpreted in amniotes.

Acrodonty, pleurodonty, and thecodonty are defined by the geometry of the attachment of a tooth to the surrounding jawbone [2], [4]. These are useful descriptors of the depth to which a tooth is implanted into the jaw and the general strength with which a tooth is fastened to the bone. From a phylogenetic perspective; however, these three categories should not be used as separate character states because they are the results of the interplay between different amounts and arrangements of periodontal tissues that can vary significantly, even within a single jaw [8], [11], [34]. Instead, interpretations of amniote tooth implantation must consider the identities of the tissues that form the periodontium, and their histological properties [3], [8], [24], [34]. This new approach can then lead to testable hypotheses of homology. Whereas the presence of cementum, alveolar bone, and periodontal ligament are not synapomorphies of any particular amniote clade, their arrangements within the alveolus may be phylogenetically informative. For example, diadectids and mosasauroid squamates share a thecodont mode of tooth implantation; however, the periodontium consists of different arrangements and types of cementum. In mosasaurs, the tooth root is composed of large quantities of vascularized, cellular cementum that is anchored to the alveolar bone by a mineralized periodontal ligament [2], [7], [34]. In diadectids, the tooth root is coated in a thin band of avascular cellular cementum, similar to mammals and crocodilians. Thecodonty in diadectids and mosasaurs thus reflect analogous forms of tooth implantation that clearly evolved independently, but the underlying developmental mechanisms are homologous, given that the tissues coating the tooth roots in both taxa are identified as forms of cementum. By comparison, the classical interpretation would be that both diadectids and mosasaurs retained the plesiomorphic condition of tooth ankylosis via a pedestal of “bone of attachment” [15], [42]. Not only would this classical interpretation be incorrect, it would mask an important characteristic of the amniote periodontium: it is a modular system, consisting of three major tissues, that has been modified to suit the functional demands of the dentition in any given taxon.

## Conclusions

Comparisons between diadectid, mammalian and crocodilian forms of thecodonty demonstrate that teeth of these three taxa were implanted and attached in similar fashions. We reject the hypothesis that tooth attachment in diadectids was through ankylosis to “bone of attachment” and emphasize that the attachment of the tooth root to the socket in diadectids is much more complex than previously thought. The tooth root is coated in successive layers of acellular and cellular cementum, which were connected to alveolar bone by a ligamentous attachment (a thecodont gomphosis) that mineralized later in ontogeny of the tooth or of the animal (Figure 10). Very little remains of the periodontal ligament in diadectids, because any soft tissue components of the periodontium would have been lost shortly after death of the animal, as was demonstrated in the thin sections of a fossil horse (Figure 3). However, dense networks of Sharpey's fibers within the alveolar bone and cementum in the diadectid specimens could only have formed as the mineralized components of a ligament within the alveolus: the periodontal ligament. Furthermore, edentulous upper and lower jaws of diadectids provide taphonomic evidence that a soft tissue attachment between the tooth roots and the alveoli must have existed at a particular point in the lifespan of each tooth. Any form of ankylosis that occurred in diadectids was thus through mineralization of the periodontal ligament and not through fusion of the tooth root to “bone of attachment”, more properly called alveolar bone.

The presence of a tripartite periodontium in diadectids supports the hypothesis that the dental follicle and its derivatives (cementum, alveolar bone, and periodontal ligament) were present in these stem amniotes. We provide the earliest record of a tripartite periodontium in a tetrapod by demonstrating its presence in the Diadectidae, a group that persisted from the Late Pennsylvanian into the Early Permian [27]. The presence of a tripartite periodontium in diadectids and several amniote taxa [2], [3], [8], [11] provides an increasing amount of evidence that all amniotes share the ability to produce the periodontal tissues that have historically been associated with mammalian and crocodilian thecodonty. Whereas many amniote taxa do not exhibit thecodonty, or a ligamentous tooth attachment (e.g. extant lepidosaurs), the alternate forms of tooth implantation are highly derived arrangements of alveolar bone and cementum [16], [41], rather than being plesiomorphic, as previously suggested [15]. The ways in which tooth implantations are defined for amniotes must be changed in order to reflect the homologies of the periodontal tissues [2], [3]. Future classifications of tooth implantation in amniotes should emphasize the amounts, arrangements, and identities of the periodontal tissues that attach a tooth to the jaw. This will be important for determining which aspects of amniote tooth implantation are functionally and phylogenetically informative.
